# Flow parsing and biological motion

**DOI:** 10.3758/s13414-020-02217-6

**Published:** 2021-02-24

**Authors:** Katja M. Mayer, Hugh Riddell, Markus Lappe

**Affiliations:** 1 Institute for Psychology, University of Muenster, Fliednerstr. 21, 48149 Muenster, Germany; 2 School of Psychology, Curtin University, Perth, Australia; 3 Otto Creutzfeld Center for Cognitive and Behavioral Neuroscience, University of Muenster, Muenster, Germany

**Keywords:** Flow parsing, Biological motion, Optic flow, Motion perception

## Abstract

Flow parsing is a way to estimate the direction of scene-relative motion of independently moving objects during self-motion of the observer. So far, this has been tested for simple geometric shapes such as dots or bars. Whether further cues such as prior knowledge about typical directions of an object’s movement, e.g., typical human motion, are considered in the estimations is currently unclear. Here, we adjudicated between the theory that the direction of scene-relative motion of humans is estimated exclusively by flow parsing, just like for simple geometric objects, and the theory that prior knowledge about biological motion affects estimation of perceived direction of scene-relative motion of humans. We placed a human point-light walker in optic flow fields that simulated forward motion of the observer. We introduced conflicts between biological features of the walker (i.e., facing and articulation) and the direction of scene-relative motion. We investigated whether perceived direction of scene-relative motion was biased towards biological features and compared the results to perceived direction of scene-relative motion of scrambled walkers and dot clouds. We found that for humans the perceived direction of scene-relative motion was biased towards biological features. Additionally, we found larger flow parsing gain for humans compared to the other walker types. This indicates that flow parsing is not the only visual mechanism relevant for estimating the direction of scene-relative motion of independently moving objects during self-motion: observers also rely on prior knowledge about typical object motion, such as typical facing and articulation of humans.

## Introduction

Extracting independent object motion in a scene during self-motion is a challenge for the visual system: any motion on the retina might be due to either the self-motion of the observer, the motion of objects in the scene, or some combination of both sources of motion (Wallach, 1987). The theory of flow parsing (Rushton & Warren, 2005) proposes that the visual system extracts scene-relative object motion by using optic flow analysis to “subtract” the retinal motion component that is due to self-motion from the full retinal motion field. Any motion remaining after this subtraction would consequently be due to the motion of objects external to the observer.

To date, many studies have demonstrated flow parsing for extracting the motion of inanimate, abstract objects during self-motion. Studies either presented optic flow fields that simulated motion of an observer (Foulkes et al., 2013; Niehorster & Li, 2017; Rogers et al., 2017; Rushton & Warren, 2005; Rushton et al., 2018; Vaina et al., 2014; Warren & Rushton, 2007, 2008, 2009a, b, 2012) or the observer physically moved (Dokka et al., 2015b, 2015a; Dupin & Wexler, 2013; Fajen et al., 2013b; Fajen & Matthis, 2013a). Concurrently, simple geometric target objects such as cubes (e.g., Dupin & Wexler, 2013) or dots (Niehorster & Li, 2017) that moved independently of the optic flow were presented. Observers performed tasks that involved detection of the motion of the target object (e.g., Rushton & Warren, 2005), judging its direction of scene-relative motion (e.g., Warren & Rushton, 2007) or estimating whether collision with the object was immanent (Fajen & Matthis, 2013a).

Further findings provided more details on flow parsing and related it to other flow-based visual mechanisms (Warren & Rushton, 2008, 2009a, b, 2012; Foulkes et al., 2013; Fajen & Matthis, 2013a, b; Niehorster & Li, 2017; Rogers et al., 2017). When estimating the direction of scene-relative motion of a dot probe embedded in different flow fields it was found that flow parsing depended on the properties of the optic flow (Warren & Rushton, 2009a). Reducing the optic flow to a small area surrounding a dot probe reduced flow parsing with respect to displays in which optic flow was present across the whole field of view but spared in the area surrounding the dot probe (Warren & Rushton, 2009a). Furthermore, it was found that flow parsing and heading estimation depended on similar properties of the optic flow field (Foulkes et al., 2013). Both processes depended on how many stationary objects defined the flow field and how much noise was added to the optic flow. Recently, the role of optic flow for flow parsing was further corroborated (Rushton et al., 2018). Form and position cues in the flow field did not contribute to flow parsing. These findings indicated that visual cues other than optic flow were irrelevant for estimating motion profiles of independently moving objects during self-motion. Regarding peripheral vision, it was found that peripherally perceived optic flow contributed to flow parsing which indicated that flow parsing is consistent with the concept of global processing of optic flow (Rogers et al., 2017).

In the brain, flow parsing might be enabled by cells in the MT/MST complex, an area involved in processing optic flow (see Lappe & van den Berg, 1999, for a review) or areas V3A and V6 (Galletti & Fattori, 2003; Pitzalis et al., 2015, 2020). Optic flow detectors in the MT/MST complex might act as filters for optic flow due to self-motion and thus provide necessary information that enabled flow parsing (Warren & Rushton, 2007). Recently, the neural substrates underlying flow parsing were located with a variety of brain mapping and behavioral techniques (Pitzalis et al., 2020). The results identified a network consisting of areas responsive to self-motion (e.g., LOR), motion of independently moving objects (e.g., MT complex) and areas responsive to both types of motion (e.g., V6 complex, V3A). A neuropsychological study suggested that the neural processes enabling flow parsing were distinct from general motion processing (Vaina et al., 2014). The study showed that flow parsing was possible even for observers who were incapable of perceiving relative motion due to brain lesions. In addition to experimental studies, a computational model was proposed to describe flow parsing. In this model, neurons in MT and MST interact to achieve flow parsing by processing local and global motion information (Layton & Fajen, 2016).

Although flow parsing has consistently been found for different viewing conditions, there is evidence that it does not account fully for the self-motion component in the retinal motion. For example, even though flow parsing robustly occurred across different experimental paradigms it never completely discounted the speed of self-motion (Niehorster & Li, 2017). The flow parsing gain, defined as the ratio to which flow parsing removed the motion component of the observer from the retinal motion (Niehorster & Li, 2017), was 0.67 or less depending on the exact viewing conditions. Furthermore, it was found that the flow parsing gain depended on whether observers were able to rely on vestibular motion cues about self-motion in addition to optic flow (Dokka et al., 2015b). Flow parsing gains ranged from 0.47 when only visual information about self-motion was available to 0.56 when visual and vestibular information about self-motion was available (Dokka et al., 2015b). Regarding visual backgrounds, flow parsing gains vary between 0.65 and 0.75 depending on whether a visual background is present (Dupin & Wexler, 2013). The highest flow parsing gain reported for visual stimuli is 0.8 (Xie et al., 2020).

There is also evidence that flow parsing can be modified by additional information. Several studies (Dupin & Wexler, 2013; Dokka et al., 2015a, b; MacNeilage et al., 2012; Fajen & Matthis, 2013a, b; Sasaki et al., 2017) showed that providing vestibular information optimized flow parsing when judging the direction of scene-relative motion of an independently moving object. In these studies, observers walked through virtual environments (Fajen & Matthis, 2013a, b), moved in front of a screen wearing a head tracker (Dupin & Wexler, 2013) or were passively moved by a moving platform (MacNeilage et al., 2012; Dokka et al., 2015b). Thus, multisensory cues about self-motion supplement optic flow for flow parsing. Yet, from a theoretical point of view, flow parsing remains a process that determines the scene-relative motion of an object by its deviation from the self-motion of the observer.

Beyond vestibular and optic-flow-related information, however, there may be sources of information which originate from the independently moving objects themselves. Previous studies that investigated flow parsing induced optic flow and concurrently presented simple independently moving objects such as dots (e.g., Rushton et al., 2018) or basic three-dimensional geometric shapes (e.g., Fajen et al., 2013b). More complex objects, however, might provide cues that help separating sources of retinal motion in addition to optic flow analyses. One of the most behaviorally relevant and frequently occurring sources of independent motions in a scene are other humans that we encounter as we move through our environment. Biological stimuli such as walking humans provide visual cues that could potentially be used to extract information about the direction of independent motion during self-motion. Such biological motion cues refer to the articulation (i.e., limb motion) and the facing (i.e., the orientation of the body).

In general, human observers have been found to use prior knowledge of human shape and motion to facilitate visual processing (Cavanagh et al., 2001; Troje & Westhoff, 2006). For example, familiarity with the human body can be used to solve ambiguous percepts. Observers used knowledge about the typical association between articulation and translation (i.e., whole body motion through space) of walking humans in order to infer self-motion (Riddell & Lappe, 2017). In that study, the articulation and translation speed of a walker were the only optic flow cues. Despite the sparse information, observers were able to infer whether self-motion was simulated or not. Moreover, perceived facing direction of a walker was biased towards the translation direction when conflicts between facing and translation direction were induced (Masselink & Lappe, 2015). This finding suggested that close associations between typical facing and translation direction were formed by extensive experience with the stimulus which helped solving ambiguous sensory input. Similarly, faster translation speeds of humans were associated with percepts of running rather than walking in stimuli in which articulation of running and walking was morphed (Thurman & Lu, 2016).

From a theoretical perspective, observers could thus supplement the observer-motion-based process of flow parsing with object-based information derived from detailed knowledge about the shape and motion of a human. The biological motion of a human walker could be a source of information for estimating the direction of scene-relative motion of that human during self-motion. Such a supplementation is of conceptual interest because optic flow analysis and biological motion perception are supported by distinct perceptual processes and brain pathways. Optic flow analysis is mathematically based on the geometry of rigid body motion (Longuet-Higgins & Prazdny, 1980) and the assumption of a rigid environment. It is thought to proceed by the analysis of retinal motion signals in the dorsal pathway of the brain (Lappe & van den Berg, 1999; Galletti & Fattori, 2003; Britten, 2008). The perception of the motion of human bodies, in contrast, does not need retinal motion signals (Beintema & Lappe, 2002) and can be understood as a sequential process of body posture analysis (Lange et al., 2006) followed by motion analysis in posture space (Theusner et al., 2014). These processes are performed by areas in the ventral stream of the visual brain (Michels et al., 2005; Singer & Sheinberg, 2010; Vangeneugden et al., 2011). Accordingly, biological motion perception and heading estimation from optic flow can be processed in parallel and do not interact with each other (Mayer et al., 2019). Whether biological motion information is used for estimating direction of human movement during self-motion is thus of theoretical interest for both understanding the mechanisms underlying estimation of direction of motion of other humans during self-motion and understanding of the motion perception pathways in general.

In the present study, we tested whether observers rely on self-motion information (i.e., flow parsing) or object information (i.e., biological motion) when estimating direction of motion of other humans during self-motion. We placed different types of point-light walkers (PLWs; Johansson, 1973) that moved in multiple directions in an optic flow field that simulated forward motion of an observer and measured the perceived direction of scene-relative motion of the walker. We chose PLWs over more natural stimuli to ensure that we only provide observers with the cues of interest for the present study: motion vectors, facing and articulation. The PLWs were a human walker (Intact), scrambled versions of the walker (Scrambled) and dot clouds (Cloud; i.e., dot clouds consisting of scrambled PLWs without the nonrigid motion caused by articulation). The Intact walker contained all cues for biological motion and was our main experimental stimulus. The Cloud walkers contained the same translational motion as the Intact walker but not its biological motion cues. It served as our main non-biological control stimulus. The Scrambled walkers were nonbiological control stimuli that are often used in studies on biological motion perception (Troje & Westhoff, 2006; Grossman et al., 2000; Lange & Lappe, 2007; Bertenthal & Pinto, 1994; Cutting et al., 1988). They display the same image motion of dots as the Intact walker but starting from different locations in space. Thus, they contain the same low-level motion cues as the Intact walker but do not convey the shape of the human body, and hence no biological motion percept. The Scrambled walkers served as a control for possible effects on flow parsing of the nonrigid motion produced by the walker. This was critical for our study because the presence of nonrigid motion of humans (and scrambled versions of human PLWs) affects optic flow perception (Riddell & Lappe, 2017; Koerfer & Lappe, 2020; Hülemeier & Lappe, 2020) and, therefore, might also affect flow parsing. For the Intact walker, we induced conflicts between biological features (i.e., facing and articulation) and the direction of scene-relative motion. This allowed us to adjudicate between two theories on how direction of scene-relative motion is estimated for biological objects (i.e., walking humans). The first theory, pure flow parsing, proposes that humans are parsed like any other geometric object, and that flow parsing is achieved directly from optic flow analysis (i.e., information originating from self-motion of the observer). In this case, we expect no difference between Intact, Scrambled and Cloud walkers. The second theory, in contrast, proposes that typical human shape and motion cues contained in biological motion act as sources of information about the direction of scene-relative motion of a human during self-motion (i.e., information originating from the independently moving object itself). In this case, we expect to find biases in the perceived direction of scene-relative motion towards biological features (i.e., articulation and facing of the human). Furthermore, we included two control conditions regarding the relative motion between the observer and the walkers. In the first one we presented the walkers in the absence of observer motion (Stationary condition) in order to test for general biases in the perception of our stimuli. In the second one, we moved the walker allying with the observer in depth so as to keep a constant distance between the two. This conditions investigated whether observers rely on the height and height change of the body of the Intact walker to solve the task. Relative retinal size and size change of the human body can serve as a valid cue when estimating a person’s location and motion in depth.

## Methods

### Participants

We conducted a power analysis using G*Power (Faul et al., 2009) to determine the optimal sample size. For selection of the parameters, we followed previous research into processing biological motion and optic flow (Riddell & Lappe, 2018; Mayer et al., 2019). To detect a small to moderate effect (${\eta _{p}^{2}}$ = .35) with *α* = .05 and a power of 80%, G*Power revealed that a sample size of 11 participants would be optimal. Thirteen persons with normal or corrected-to-normal vision (mean age = 22 years, standard deviation = 6 years, all right-handed, eight females), recruited from the University of Muenster, volunteered. Participation was compensated with course credit. They were informed that they would participate in a study investigating optic flow and the motion of other beings, but were naive with respect to specific hypotheses. They gave written informed consent prior to participating. Ethics were approved by the local ethics committee of the Department for Psychology and Sports Sciences of the University of Muenster. Data of one participant were excluded from the analyses due to potential misunderstanding of task instructions.

### Setup

Experiments took place in a darkened and quiet laboratory. Participants were seated in a chair 100 cm from a 248 cm ×200 cm screen. Stimuli were back-projected onto the screen by a VDC Display Systems Marquee 8500 projector (resolution: 800 pixels × 600 pixels; refresh rate: 60 Hz). Experiments were presented with an Apple Mac Book Pro with an Intel Iris Pro built-in graphics card running Matlab and the Psychtoolbox (Kleiner et al., 2007) as well as the OpenGL libraries (version 2.1). Responses were collected using the left and right arrow keys and the space bar of a standard Apple keyboard.

### Stimuli

**Fig. 1:**
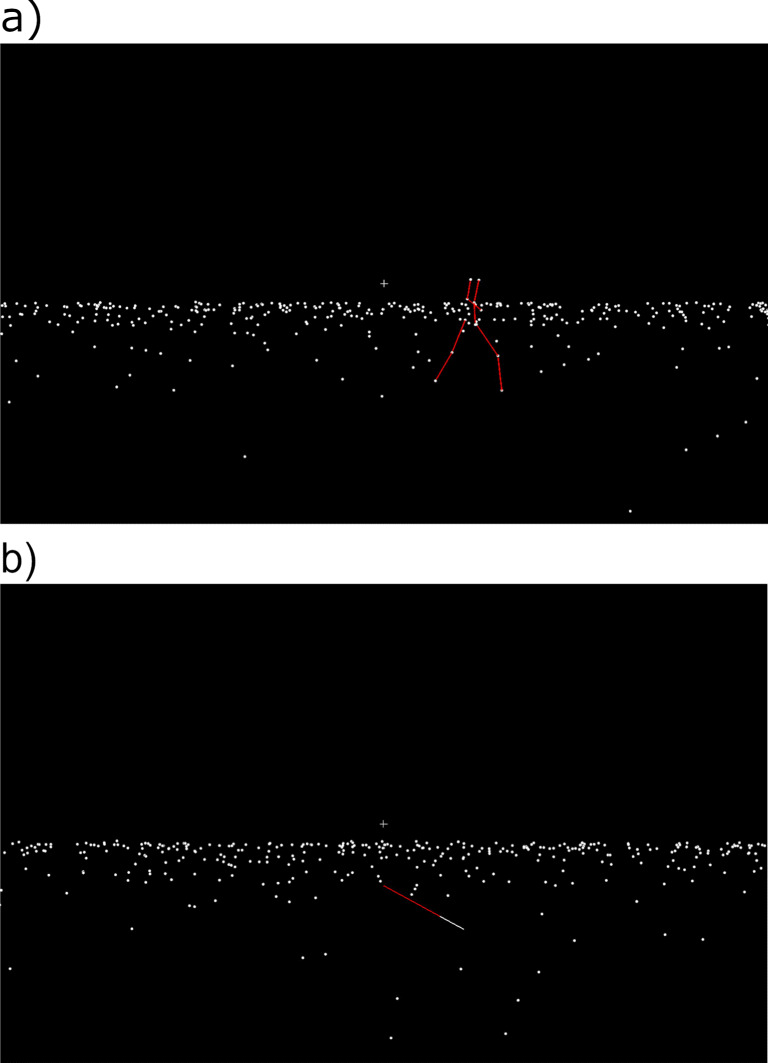
**a** One frame of the animation displaying an example of the Intact condition. An intact human point-light walker translated across a plane defined by dots. The lines are for illustration purposes and were not shown in the experiment. **b** Response line that was fixed in the dot plane by its red (*dark gray*) end and could be rotated in order to indicate the perceived direction of scene-relative motion of the walker across the plane

Stimuli were scenes displaying a walker, a ground plane covered by random dots, and a fixation cross (Fig. 1a). The scene was set up as if viewed through a virtual camera (see Riddell & Lappe, 2017; Mayer et al., 2019). The location of the camera was defined as the origin of a coordinate system (i.e., the eyes of the observer) used to describe the scene. The *x*-axis expanded from left to right, the *y*-axis from top to bottom and the *z*-axis expanded in depth. Forward motion of the observer was simulated by moving the camera through the scene along the *z*-axis. The scene was rendered against a black background and subtended 196 cm (89^∘^ visual angle) in height and 248 cm (102.2^∘^ visual angle) in width.

At the center of the screen, there was a white fixation cross (5 cm ×5 cm; 2.9^∘^ × 2.9^∘^ visual angle) which remained visible throughout each trial. The ground plane was defined by 350 white dots (0.74^∘^ visual angle in diameter) randomly distributed across the plane. On half of the trials, forward motion of the observer was simulated by moving the camera across the ground plane along the *z*-axis at 2 m/s. Three walker types were used: Intact, Scrambled, and Cloud. The Intact walker consisted of 12 white dots (0.74^∘^ visual angle in diameter) that were recorded from a male human walking at 1.2 m/s (de Lussanet et al., 2008; Riddell & Lappe, 2017). The dots marked shoulders, elbows, wrists, hips, knees and ankles. The PLW expanded to a maximum size of 3.4^∘^ visual angle in width at the hip (note that the expansion at the feet deviated depending on the articulation phase) and 15.9^∘^ visual angle in height. The Intact walker faced to the right-hand side of the screen in all conditions, thus the observers saw the profile of the walker. Five scrambled versions of the Intact walker were created (Scrambled). The Scrambled walkers consisted of the same number of dots. Scrambling was generated by spatially randomizing the *x*- and the *y*-coordinates of the dots defining the PLW but keeping the *z*-coordinate and the articulation parameters intact. Scrambling of the *x*- and *y*-coordinates was constrained to the maximal expansion of the intact PLW during articulation. Depending on the articulation phase, the scrambled PLWs maximally expanded 10.3^∘^ visual angle in width (i.e., approximately the maximum expansion of the feet during the articulation of the Intact walker) and 15.4^∘^ visual angle in height. The Cloud walkers consisted of the same number of dots but in fixed random locations without any independent dot motion. They were created by removing the articulation parameters from the Scrambled walkers but keeping the *x*-, *y*- and *z*-coordinates of the dots. Each of the five Scrambled and each of the five Cloud walkers were presented in a pseudo-random order ensuring that each walker was presented equally often in the experiment.

**Fig. 2:**
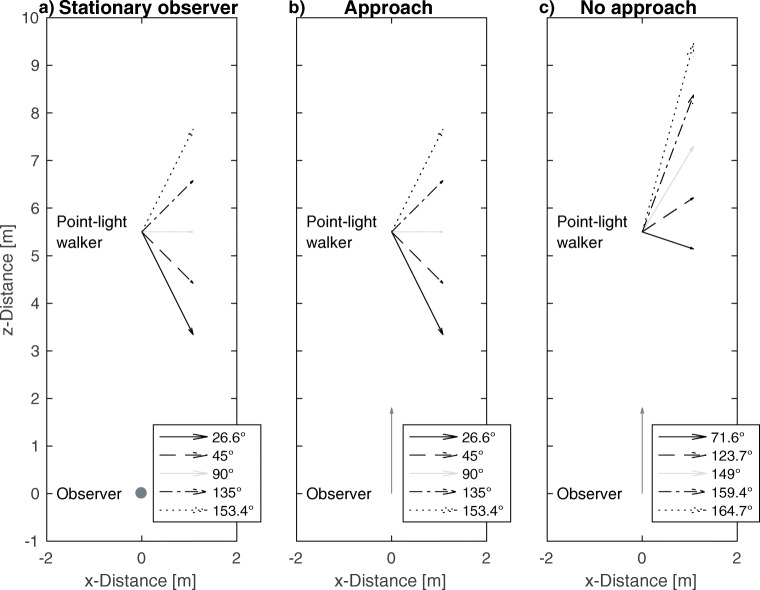
The scene shown in the experiment from the bird’s eye perspective. Arrows indicate motion vectors of the walkers (black, dashed, light gray, dash-dotted and dotted arrows) and the observer (dark gray arrows). The dark gray dot marks a stationary observer. In the No approach condition, the motion vector of the observer was added to the motion vectors of the point-light walker in the Approach condition

**Fig. 3:**
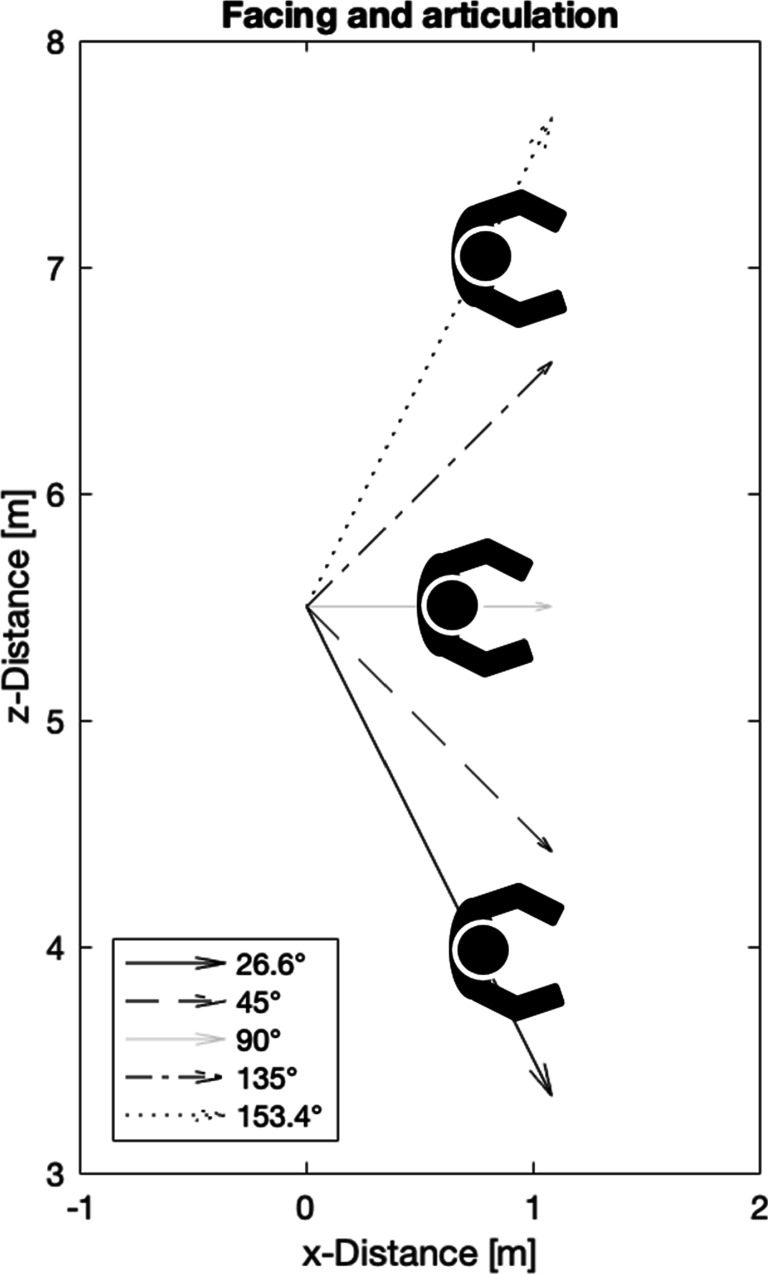
The scene viewed from bird’s eye perspective. The arrows indicate the directions of scene-relative motion of the walkers. Across all directions of scene-relative motion, the Intact walker faced and articulated to the right which means that observers saw the profile of the walker in all conditions

All independent motion stimuli translated to the right-hand side of the screen along different directions (Fig. 2). The directions of scene-relative motion were defined with respect to rotation about a *y*-axis (0^∘^ was the direction directly along the *z*-axis, opposite to the forward movement of the observer; 90^∘^ was the direction directly along the *x*-axis, perpendicular to the forward movement of the observer; 180^∘^ was the direction directly along the *z*-direction in the same direction as the forward movement of the observer). Five directions of scene-relative motion were selected for the experiment. Two directions were towards the observer (26.6^∘^ and 45^∘^), one was to the right of the screen (90^∘^) and two were away from the observer (135^∘^ and 153.4^∘^). Except for the 90^∘^ condition, there was a conflict between the biological features of the Intact walker (facing direction and articulation) and the direction of scene-relative motion because the biological features of the Intact walker always faced and articulated to the right (90^∘^) irrespective of the direction of scene-relative motion (Fig. 3).

Introducing conflicts allowed us to study the influence of one particular facing and articulation direction on different directions of scene-relative motion. We chose this approach over the complementary approach of combining a fixed direction of scene-relative motion with different facing direction because facing directions other than 90^∘^ are known to introduce ambiguities and bistability in biological motion perception (Vanrie et al., 2004; Theusner et al., 2014; de Lussanet & Lappe, 2012) that may confound the results.

Directions of scene-relative motion without observer motion are shown in Fig. 2a. For conditions with simulated observer motion two combinations of observer and walker translation were tested. In the Approach condition, the observer naturally approached the walker during trials in which forward motion of the observer was simulated (Fig. 2b). Thus, the walker’s relative size grew as the observer moved forward and the walker moved towards the observer. Relative size of a familiar object can provide information about its location in depth and therefore affect the estimate of the direction of scene-relative motion. We included the No approach condition in which we added the motion vector of the observer (2 m/s in *z*-direction) to the direction of scene-relative motion of the walker. Thus, in this condition the relative size of the walker remained constant throughout a trial (Fig. 2c). This condition resembled the situation in which a person walked in front of a moving observer and kept a constant distance from the observer. The No approach condition is comparable to the majority of experiments on flow parsing up to now (e.g., Warren & Rushton, 2008; Foulkes et al., 2013; Rushton et al., 2018), in which a singular dot is displayed against an optic flow field, but is separate from the field (i.e. the dot does not expand with the flow field). The Approach condition, on the other hand, more accurately reflects natural viewing conditions in which an observer approaches the moving object, and serves as a means to measure how observers process object movement (specifically biological motion) in a naturalistic setting. Example videos of the stimuli can be found at https://www.doi.org/10.6084/m9.figshare.13143206.

### Task

Participants reported the perceived direction of scene-relative motion of the walker on the ground plane. They did so by adjusting a red line with a white tip that appeared on the ground plane at the end of the trial (Fig. 1b). The red end of the line was fixed at the starting location of the walker. The line could be rotated 360^∘^ on the plane about that end. The original orientation of the line was randomly chosen for every trial between 0^∘^ (pointing directly towards the observer) and 180^∘^ (pointing away from the observer). Participants adjusted the line by using the left and right arrow keys of a standard Apple keyboard.

### Design

The experiment was set up as a 2 (Observer motion: Heading, Stationary) × 2 (Relative size: Approach, No approach) × 3 (Walker: Intact, Cloud, Scrambled) × 5 (Directions of scene-relative motion: 26.6^∘^, 45^∘^, 90^∘^, 135^∘^, 153.4^∘^ for Stationary and Approach; 71.6^∘^, 123.7^∘^, 149^∘^, 159.4^∘^, 164.7^∘^ for No approach) design with repeated measures on all factors resulting in 60 conditions.

### Procedure

Prior to the experiment, participants were informed that their task was to estimate the direction of scene-relative motion of a walker. They were asked to fixate the cross presented at the center of the screen throughout a trial, but head movements were not constrained and eye movements were not tracked. A few practice trials were presented before the actual experiment.

Before the first trial, the fixation cross was shown for 1 s. It remained visible throughout the trial. Every trial started with the appearance of the ground plane and the walker. At the beginning of a trial, the walker appeared at the center of the screen, 5.5 m in front of the observer. On a given trial, the walker translated to the right of the screen along one out of five directions (Fig. 2). After 60 frames (1 s) the walker disappeared but the ground plane and the fixation cross remained visible. In trials with simulated forward movement of the observer the movement was stopped leaving a stationary ground plane. A red line with a white tip appeared on the ground plane and participants used the left and right arrow keys to adjust the line in a way that indicated the perceived direction of scene-relative motion of the walker over the ground plane. When they had finished adjusting they pressed the space bar to record a response. There was no time limit to enter the response. Once the space bar was pressed an inter-trial interval consisting of a white fixation cross rendered against a black background was shown for 1 s. There was a self-timed break after every set of 50 trials.

Each condition was shown five times leading to 300 trials in total. Conditions were presented in randomized order. In total, the experiment took approximately 30 min.

### Data analyses

In the absence of observer motion (Stationary condition), Approach and No approach trials were physically identical. We therefore collapsed across Approach and No approach trials when the observer was stationary for all analyses.

We tested the perceived directions of scene-relative motion using repeated measures ANOVAs. We analyzed the Stationary, the Approach and the No approach condition separately. Also, we separately analyzed the comparisons between the Intact and the Cloud walker and between the Intact and the Scrambled walker. Thus, our data analyses were set up as 2 (Walker: Intact, Cloud; or Intact, Scrambled) × 5 (Direction of scene-relative motion: 26.6^∘^, 45^∘^, 90^∘^, 135^∘^, 153.4^∘^ for Stationary and Approach; 71.6^∘^, 123.7^∘^, 149^∘^, 159.4^∘^, 164.7^∘^ for No approach) repeated measures ANOVAs. We accepted main effects and interactions as significant if *p*<.05. When appropriate, we applied Benjamini-Hochberg correction for multiple comparisons with a false discovery rate of 0.25.

In order to test for influences of relative size of a walker during observer motion, we included additional 2 (Relative size: Approach, No approach) × 2 (Walker: Intact, Cloud; or Intact, Scrambled) repeated measures ANOVAs.

Furthermore, we calculated the flow parsing gain. We conducted the analysis of the flow parsing gain in the 90^∘^ condition, i.e., the condition with no conflict between biological features and direction of scene-relative motion for the Intact walker. In this condition the direction of scene-relative motion of the observer is orthogonal to that of the walkers. Thus, the proportion to which an observer compensates for their own self-motion directly relates to the angle of the perceived direction of scene-relative motion of the walker. We defined flow parsing gain as the proportion of self-motion in *z*-direction that participants subtracted from the perceived translation of the walker (e.g., Niehorster & Li, 2017). We tested whether the flow parsing gain of the Intact walker was larger than the flow parsing gains of the Scrambled and the Cloud walkers.

### Hypotheses

If human walkers are parsed like any other geometric object, we expect consistent perceived directions of scene-relative motion for Intact, Scrambled and Cloud walkers. If, however, observers rely on biological features to estimate direction of scene-relative motion during self-motion for other humans we expect an interaction between the direction of scene-relative motion and the walker type. For the Intact walker, responses would be biased towards 90^∘^, i.e., the direction of the biological features. Specifically, if the observer indicates a perceived direction < 90^∘^ for Scrambled and Cloud walkers we expect larger angles for the condition with the Intact walker. If the observer indicates a perceived direction > 90^∘^ for Scrambled and Cloud walkers we expect smaller angles for the condition with the Intact walker. If observers rely entirely on biological features of the Intact walker the perceived direction of scene-relative motion would always be 90^∘^.

As the nonrigid motion of the Intact walker disturbs optic flow necessary for estimating self-motion (Riddell & Lappe, 2017; Hülemeier & Lappe, 2020) it could be a potential source of uncertainty for flow parsing estimates. To investigate whether nonrigid motion of a walker affected flow parsing we first tested for differences between the Intact and the Cloud (i.e., a non-human stimulus without nonrigid motion) walkers and then for differences between the Intact and the Scrambled (i.e., a non-human stimulus with the same nonrigid motion as the Intact walker) walkers.

Apart from typical associations between facing, articulation and direction of motion, observers are familiar with the height of humans. Changes in relative size due to self-motion or motion of another human provide cues about location of the other human in the scene. If observers rely predominantly on relative size of the Intact walker in order to estimate direction of scene-relative motion, we expect these biases to only be present in the Approach condition as relative size was controlled for in the No approach condition. Biases due to relative size would be reflected in an interaction between Walker and Relative size which would be driven by differences between the two walker types in the Approach condition that are absent in the No approach condition.

A further hypothesis concerns the flow parsing gain in the 90^∘^ condition. For the Intact walker translating along the 90^∘^ direction in the scene, there are no conflicts between biological features and the direction of scene-relative motion. Thus, the three walker types only differ with respect to biological stimulus information on direction of scene-relative motion. Observers, therefore, could combine biological features and flow parsing and might be able to increase flow parsing gain for the Intact walker. In this case, the flow parsing gain for the Intact walker is expected to exceed the flow parsing gains for the Scrambled and the Cloud walkers. Furthermore, this could lead to flow parsing gains larger than the previously reported ones for inanimate objects (approximately 0.8 for fully visual stimuli, Xie et al., 2020). If observers are capable of perfect flow parsing (i.e., a flow parsing gain of 1) we expect to find perceived directions of scene-relative motion to be identical to the veridical directions of scene-relative motion (Fig. 2b and c).

In addition, from the Stationary condition we explored how the biological features affected perceived direction of scene-relative motion with respect to non-human stimuli without observer motion. We compared perceived direction of scene-relative motion of the Intact walker to the Scrambled and the Cloud walker, respectively, in the absence of self-motion.

## Results

### Perceived directions of scene-relative motion

#### Intact vs Cloud walker

**Fig. 4:**
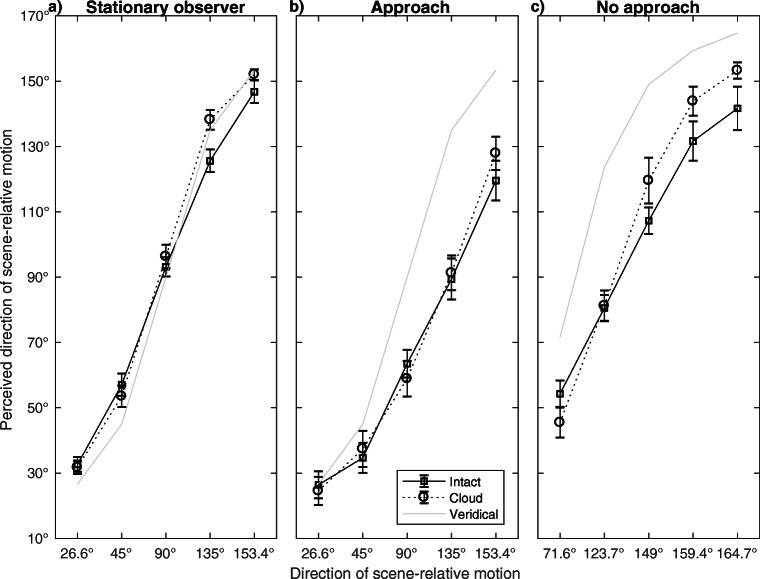
Perceived directions of scene-relative motion for the Intact and the Cloud walker. 0^∘^ is motion of the walker towards the observer, 90^∘^ is motion of the walker to the right, 180^∘^ is motion of the walker away from the observer. Error bars are +/-1 standard error of the means

For Stationary observers (Fig. 4a), we found an interaction between Walker and Direction of scene-relative motion (*F*(2.23, 24.48) = 9.77, *p* = .001, Greenhouse-Geisser corrected, ${\eta _{p}^{2}}$ = .47) and a main effect of Direction of scene-relative motion (*F*(2.08, 22.84) = 398.96, *p*<.001, Greenhouse-Geisser corrected, ${\eta _{p}^{2}}$ = .97). The main effect of Walker was not significant (*F*(1, 11) = 3.83, *p* = .08, *ns*). For the Approach condition (Fig. 4b), we found an interaction between Walker and Direction of scene-relative motion (*F*(4, 44) = 3.68, *p* = .01, ${\eta _{p}^{2}}$ = .25) and a main effect of Direction of scene-relative motion (*F*(1.40, 15.41) = 62.61, *p*<.001, Greenhouse-Geisser corrected, ${\eta _{p}^{2}}$ = .85). The main effect of Walker was not significant (*F*(1, 11) = 0.51, *p* = .49, *ns*). For the No approach condition (Fig. 4c), we found an interaction between Walker and Direction of scene-relative motion (*F*(1.87, 20.62) = 5.52, *p* = .01, Greenhouse-Geisser corrected, ${\eta _{p}^{2}}$ = .33). The main effects of Direction of scene-relative motion (*F*(1.59, 17.44) = 103.55, *p*<.001, ${\eta _{p}^{2}}$ = .90) and Walker (*F*(1, 11) = 12.23, *p* = .01, ${\eta _{p}^{2}}$ = .53; perceived directions of scene-relative motion were smaller for the Intact walker) were also significant.

The Relative size × Walker repeated measures ANOVA revealed an interaction between Relative size and Walker (*F*(1, 11) = 7.92, *p* = .02, ${\eta _{p}^{2}}$ = .42). Importantly, this interaction was driven by differences between the two walker types in the No approach condition (planned comparison paired-samples *t*-test, Benjamini-Hochberg corrected; No approach: *t*(11) = 3.50, *p* = .01; Approach: *t*(11) = 0.71, *p* = .49). This means that Relative size had no influence on perceived direction of scene-relative motion when it was a valid cue (Approach condition). When Relative size was consistent across all directions of scene-relative motion (No approach) perceived directions of scene-relative motion were smaller (i.e., biased towards 90^∘^) for the Intact walker. No such bias was present for the Cloud walker. There was a main effects of Walker (*F*(1, 11) = 5.03, *p* = .05, ${\eta _{p}^{2}}$ = .31; perceived directions of scene-relative motion were smaller for the Intact walker). The main effect of Relative size (*F*(1, 11) = 197.43, *p*<.001, ${\eta _{p}^{2}}$ = .95) was expected because larger angles were presented in the No approach (71.6^∘^ to 164.7^∘^) than in the Approach condition (26.6^∘^ to 153.4^∘^).

#### Intact vs Scrambled walker

**Fig. 5:**
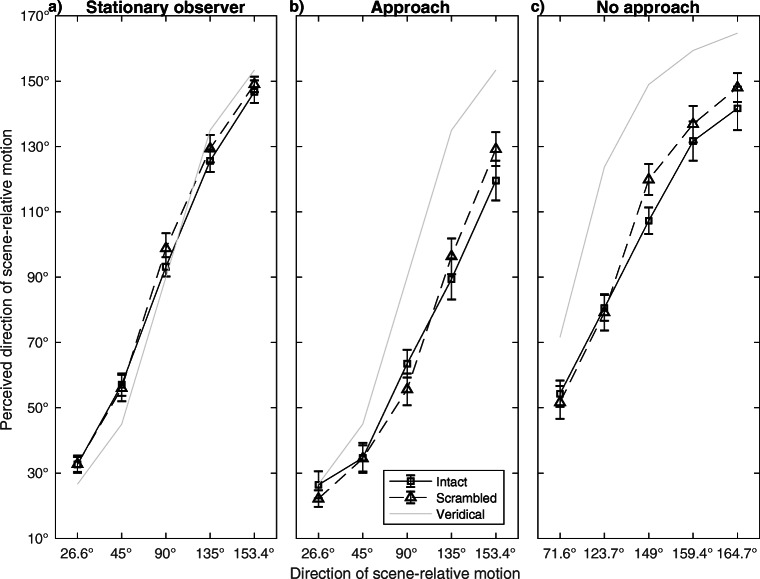
Perceived directions of scene-relative motion for the Intact and the Scrambled walker. 0^∘^ is motion of the walker towards the observer, 90^∘^ is motion of the walker to the right, 180^∘^ is motion of the walker away from the observer. Error bars are +/-1 standard error of the means

For Stationary observers (Fig. 5a), we found a main effect of Direction of scene-relative motion (*F*(4, 44) = 342.52, *p*<.001, ${\eta _{p}^{2}}$ = .97). The interaction between Direction of scene-relative motion and Walker (*F*(4, 44) = 1.13, *p* = .36, *ns*) and the main effect of Walker (*F*(1, 11) = 1.06, *p* = .33, *ns*) were not significant. For the Approach condition (Fig. 5b), we found an interaction between Direction of scene-relative motion and Walker (*F*(4, 44) = 6.47, *p*<.001, ${\eta _{p}^{2}}$ = .37) and a main effect of Direction of scene-relative motion (*F*(1.49, 16.43) = 80.55, *p*<.001, Greenhouse-Geisser corrected, ${\eta _{p}^{2}}$ = .88). The main effect of Walker was not significant (*F*(1, 11) = 0.17, *p* = .69, *ns*). For the No approach condition (Fig. 5c), we found an interaction between Direction of scene-relative motion and Walker (*F*(4, 44) = 2.89, *p* = .03, ${\eta _{p}^{2}}$ = .21). There were also main effects of Direction of scene-relative motion (*F*(1.57, 17.29) = 84.62, *p*<.001, Greenhouse-Geisser corrected, ${\eta _{p}^{2}}$ = .89) and Walker (*F*(1, 11) = 8.60, *p* = .01, ${\eta _{p}^{2}}$ = .44; perceived directions of scene-relative motion were smaller for the Intact walker).

The Relative size × Walker repeated measures ANOVA revealed a main effect of Relative size. This main effect was expected because larger angles were presented in the No approach condition than in the Approach condition (*F*(1, 11) = 272.42, *p*<.001, ${\eta _{p}^{2}}$ = .96). Relative size and Walker did not interact (*F*(1, 11) = 2.78, *p* = .12, *ns*). There was no main effect of Walker (*F*(1, 11) = 2.66, *p* = .13, *ns*).

### Flow parsing gain

**Fig. 6:**
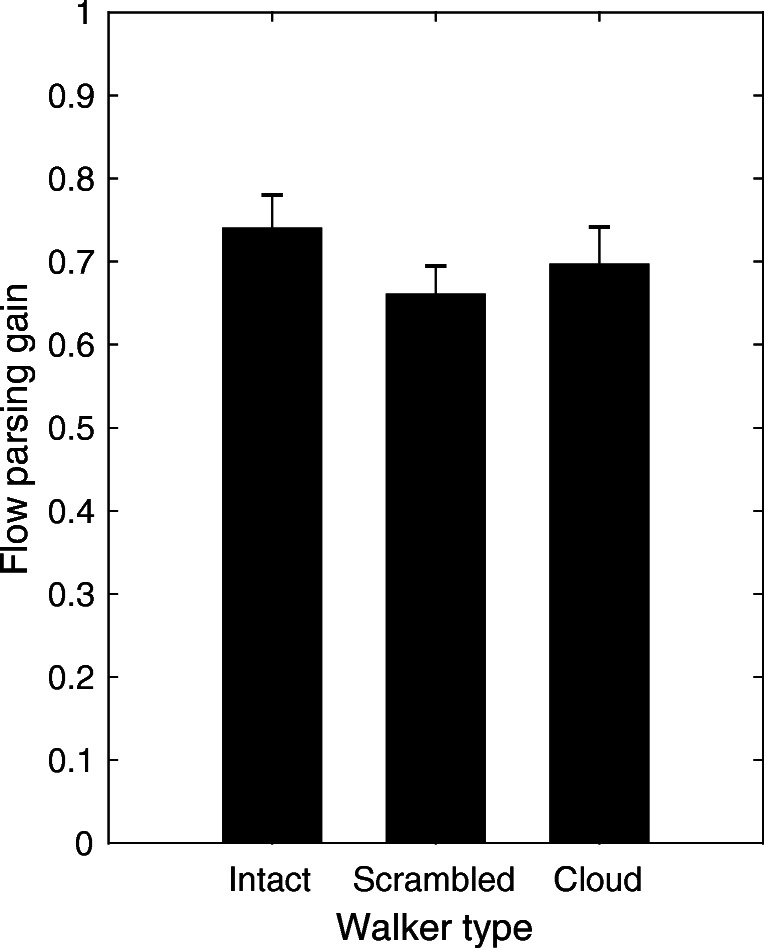
Flow parsing gains for the 90^∘^ direction of scene-relative motion (there were no conflicts between biological features and direction of scene-relative motion for the Intact walker). Error bars are +/-1 standard error of the means

Flow parsing gains were based on the 90^∘^ (Approach) condition as there was no conflict for the Intact walker between biological features and direction of scene-relative motion. Paired-samples *t*-tests revealed that the flow parsing gain for the Intact walker was larger than for the Scrambled (*t*(11) = 2.59, *p* = .03, Benjamini-Hochberg corrected) and the Cloud walker (*t*(11) = 2.32, *p* = .04, Benjamini-Hochberg corrected). The flow parsing gains are displayed in Fig. 6.

## Discussion

In the present study, we adjudicated between two theories that describe how direction of scene-relative motion of other humans during self-motion is estimated. The first theory postulates that direction of scene-relative motion is based on self-motion information (i.e., flow parsing). The second theory postulates that direction of scene-relative motion of humans is based on object features (i.e., biological features: knowledge about facing and articulation and the typically associated direction of motion). Presenting human (Intact condition) and non-human (Scrambled and Cloud condition) PLWs in optic flow fields revealed that during simulated self-motion, the perceived direction of scene-relative motion was biased towards the biological features for the Intact walker. This effect was found even when relative size of the Intact walker was controlled for (No approach condition) and could not be explained by disturbances of the optic flow field caused by the nonrigid motion of the Intact walker. Importantly, flow parsing gain (i.e., the proportion of self-motion that observers subtracted from the overall scene motion) was larger for the Intact than the Cloud and the Scrambled walkers.

From a theoretical perspective, differences between walker types are inconsistent with a pure flow parsing concept as found for geometric objects (e.g., Rushton & Warren, 2005). However, estimation of scene-relative motion was also not exclusively determined by the biological features of the Intact walker. Our data are consistent with the concept of a neural system relying on both self-motion and object information when estimating direction of scene-relative motion of other humans.

### Biases towards biological features

When comparing flow parsing of the Intact walker to the non-human walkers we expected an overestimation for directions < 90^∘^ and an underestimation for directions > 90^∘^ for the Intact walker based on biological features but not for non-human walkers. Overall, this effect is clearly seen for directions of scene-relative motion > 90^∘^ in Figs. 4 and 5 but is less clear for directions < 90^∘^. This asymmetry may originate from the exact viewing conditions presented in the present experiment. First, for directions of scene-relative motion > 90^∘^, the walkers translated away from the observer. It could be that the conflict between biological features and direction of scene-relative motion was more salient than when the walkers approached the observers which could have led to the lack of biases towards 90^∘^ for directions of scene-relative motion < 90^∘^. Second, conflicts between biological features and directions of scene-relative motion were physically strongest for 159.4^∘^ and 164.7^∘^ (No approach condition) which could lead to stronger biases towards biological features than for conditions with less salient conflict.

### Stationary observers

For Stationary observers, perceived direction of scene-relative motion was biased towards the biological features for the Intact walker when compared to the Cloud walkers. No such effect was found for the comparison between the Intact and the Scrambled walkers. The only difference between the two comparisons was the absence of nonrigid motion in the Cloud walkers. Previously, it was found that information about walking direction can even be extracted from scrambled versions of PLWs (Troje & Westhoff, 2006). Residual information about walking direction provided by nonrigid motion of the Scrambled walker could explain why we found differences between the Intact and the Cloud walkers but not between the Intact and the Scrambled walkers when the observer was stationary. Although facing information was absent in our Scrambled walkers the nonrigid motion of the point lights might still have provided enough evidence for their direction of scene-relative motion (i.e., movement towards 90^∘^).

### Relative size

Previous studies into flow parsing used simple geometric stimuli such as dots in which relative size of the independently moving object was unavailable during self-motion (e.g., Rushton et al., 2018). For the Intact walker used in the present study, however, relative size was a valid cue for estimating the direction of scene-relative motion as observers are familiar with the approximate height of other humans. To test whether observers relied on relative size, we included a condition in which relative size was available (Approach condition) and a condition in which we equated relative size across all directions of scene-relative motion (No approach condition). Comparisons between the Approach and the No approach condition for the different walker types provided no evidence for biases of the perceived directions of scene-relative motion due to relative size. There were no differences between the Intact and the non-human walkers for the Approach condition (i.e., the condition in which relative size was provided as a valid cue). It is therefore unlikely that observers predominately relied on relative size of the Intact walker in order to estimate direction of scene-relative motion. We rather assume that any differences found between the Intact walker and the non-human walkers originated from biases towards biological features.

In addition to testing whether relative body size of the Intact walker affected direction of scene-relative motion, the Relative size x Walker interaction also revealed that biases towards biological features were stronger in the No approach condition compared to the Approach condition. Stronger biases originated from systematic differences in the stimuli: larger angles were presented in the No approach condition compared to the Approach condition.

### Nonrigid motion

Flow parsing relies on optic flow and independently moving objects cause disturbances to the optic flow (Warren & Saunders, 1995). Just like rigidly moving objects, nonrigid motion originating from the articulating movements of humans can disturb optic flow. It may be that disturbances to the optic flow field could systematically bias perceived directions of scene-relative motion. To investigate whether such biases occurred in our study, we compared perceived directions of scene-relative motion of the Intact walker to both Cloud and Scrambled walkers. As the Scrambled walkers were equipped with the same nonrigid motion as the Intact walker, both types of walkers were expected to disturb the optic flow in the same way. Overall, a qualitatively consistent pattern of results was found for the comparison between the Intact and the Cloud walkers and the comparison between the Intact and the Scrambled walkers during simulated self-motion. It is therefore unlikely that biases in perceived direction of scene-relative motion originated from disturbances in the optic flow caused by nonrigid motion of the walkers but rather due to processing biological features of the Intact walker in our task.

### Implications for flow parsing

We found that object information (i.e., biological features) affected perceived direction of scene-relative motion. This finding is in line with modulation of flow parsing when multisensory object cues were available such as spatially congruent sounds (Calabro et al., 2011). Our findings show that other visual object features can affect perceived direction of scene-relative motion. Specifically, observers did not only subtract optic flow due to self-motion from the overall retinal motion, but optimized their judgments with information provided by biological features.

Although flow parsing was found to be a robust visual mechanism across many studies, previous research found that flow parsing was incomplete. That is, observers did not account for the full self-motion component of the total retinal flow (Niehorster & Li, 2017) such that flow parsing gains ranged from 0.47 (Dokka et al., 2015b) to 0.8 (Xie et al., 2020). In the present study, the flow parsing gains ranged from 0.66 in the Scrambled condition to 0.74 in the Intact condition. Thus, the flow parsing gains roughly matched the ones reported previously. Incomplete flow parsing led to the underestimation of the angles of perceived direction of scene-relative motion in the Approach and No approach conditions.

Consistent with a concept that includes biological motion processing in addition to flow parsing when estimating direction of scene-relative motion of other humans we found larger flow parsing gain for the Intact walker compared to the Scrambled and the Cloud walkers. Participants may have been able to achieve larger flow parsing gains for the Intact walker because they relied on two sources of information: biological features and optic flow. Nevertheless, the flow parsing gains implied that observers did not account for the whole self-motion component even when optic flow and biological features were available. Critically, flow parsing gain largely depends on the exact viewing conditions (Niehorster & Li, 2017). Removal of local motion of the flow field decreases the flow parsing gain (see also Warren & Rushton, 2009a). The viewing conditions in our study differed substantially from the ones of previous studies (e.g., Niehorster & Li, 2017; Warren & Rushton, 2009a). We provided optic flow from a ground plane and, in the Approach condition, from the walker itself (Riddell & Lappe, 2017). This means that optic flow was predominantly available in the bottom hemifield to mimic walking across a large field, for example. Previous research showed reduced flow parsing when providing optic flow in the lower hemifield and placing an independently moving object in the upper hemifield with respect to a condition in which optic flow was provided across the whole scene (Warren & Rushton, 2009a). Future research is necessary to test whether imbedding the walkers used in our study in a full flow field could increase the flow parsing gain even further.

### Implications for biological motion processing

In the absence of self-motion, interactions between biological features of humans and the direction of scene-relative motion have been reported previously. Perceived facing of a human PLW was biased towards the direction of scene-relative motion when the walker was shown for as little as 200 ms (Masselink & Lappe, 2015). Our findings further support the interaction between perception of biological features and perceived direction of scene-relative motion. In contrast to the previous study (Masselink & Lappe, 2015), however, our results show that biological features affected perceived direction of scene-relative motion instead of vice versa. Taking the present and the previous (Masselink & Lappe, 2015) studies together the results suggest that observers rely on prior knowledge about typical associations between biological features and direction of scene-relative motion when estimating movement of other humans. In real-life situations, this might be beneficial because it could enable observers to optimize estimates on movements of others when heading through crowds, for example (Riddell & Lappe, 2018).

### Implications for concurrent processing of optic flow and biological motion and the underlying neural correlates

Flow parsing relies on the interpretation of optic flow (e.g., Rushton & Warren, 2005). The same is true for perception of heading (Cutting et al., 1992; Lappe & van den Berg, 1999; Gibson, 1950). Differences and similarities of heading and flow parsing were investigated before (Warren et al., 2012; Rushton et al., 2018; Foulkes et al., 2013). Relating both optic flow-based processes to biological motion processing can provide insights into the underlying neural mechanisms of estimating direction of scene-relative motion during self-motion. In a previous study (Mayer et al., 2019), we used a dual-task paradigm in which we placed a human PLW in an optic flow field that simulated leftward or rightward motion of the observer. Observers judged the articulation (i.e., forward or backward articulation) of the PLW concurrently with the simulated heading direction. We found that observers were able to independently estimate heading direction and articulation of the PLW. In the present study, by comparison, we found evidence for integrated flow parsing and biological motion processing implying crosstalk between the neural substrates underlying both processes.

Taking the findings of our previous (Mayer et al., 2019) and the present study together they provide important insights into how the brain might achieve estimating of direction of scene-relative motion of other humans during self-motion. Our data are consistent with a two-staged process that requires crosstalk between areas in the ventral and the dorsal pathway. Just like found for heading (Mayer et al., 2019), flow parsing of walking humans might first be derived from optic flow independently of the processing of biological features; a procedure analogues to flow parsing of geometric objects. Only later at a second stage flow parsing might be optimized towards a plausible direction based on biological features. Candidate brain areas that could be involved in this two-staged process are MT complex, LOR, V6 and V3A as recently identified in a study into flow parsing (Pitzalis et al., 2020) for the first stage. For the second stage, the aforementioned areas might receive information from biological-motion responsive areas like STS (Grossman et al., 2000) about facing and articulation.

## Conclusions

Our results revealed that observers rely on both self-motion information provided by optic flow (i.e., flow parsing) and object information (i.e., biological features such as facing and articulation in association with the typical direction of motion) when estimating direction of motion of other humans during self-motion.
